# Ecophysiological Modeling of Grapevine Water Stress in Burgundy Terroirs by a Machine-Learning Approach

**DOI:** 10.3389/fpls.2016.00796

**Published:** 2016-06-07

**Authors:** Luca Brillante, Olivier Mathieu, Jean Lévêque, Benjamin Bois

**Affiliations:** ^1^Viticulture Research Center, Council for Agricultural Research and EconomicsConegliano, Italy; ^2^UMR CNRS/uB 6282 Biogéosciences, Université de BourgogneDijon, France; ^3^Institut Universitaire de la Vigne et du Vin “Jules Guyot,” Université de BourgogneDijon, France

**Keywords:** water stress, grapevine (*Vitis vinifera* L.), machine-learning, gradient boosting machine (GBM), water balance, carbon isotope discrimination δ^13^C, temperature, plant-soil water relationships

## Abstract

In a climate change scenario, successful modeling of the relationships between plant-soil-meteorology is crucial for a sustainable agricultural production, especially for perennial crops. Grapevines (*Vitis vinifera* L. cv Chardonnay) located in eight experimental plots (Burgundy, France) along a hillslope were monitored weekly for 3 years for leaf water potentials, both at predawn (Ψ_pd_) and at midday (Ψ_stem_). The water stress experienced by grapevine was modeled as a function of meteorological data (minimum and maximum temperature, rainfall) and soil characteristics (soil texture, gravel content, slope) by a gradient boosting machine. Model performance was assessed by comparison with carbon isotope discrimination (δ^13^C) of grape sugars at harvest and by the use of a test-set. The developed models reached outstanding prediction performance (RMSE < 0.08 MPa for Ψ_stem_ and < 0.06 MPa for Ψ_pd_), comparable to measurement accuracy. Model predictions at a daily time step improved correlation with δ^13^C data, respect to the observed trend at a weekly time scale. The role of each predictor in these models was described in order to understand how temperature, rainfall, soil texture, gravel content and slope affect the grapevine water status in the studied context. This work proposes a straight-forward strategy to simulate plant water stress in field condition, at a local scale; to investigate ecological relationships in the vineyard and adapt cultural practices to future conditions.

## Introduction

Seventy percent of the available fresh water of the world is used for agricultural purposes (FAO, 2015), and it is therefore in that field that the largest water savings can be made. Water optimization (i.e., water saving without compromising crop yield and quality) can be achieved through better infrastructure and through an in-depth understanding of the plant physiological responses to irrigation and cultural practices.

Water status is a key component in the *terroir* effect, a very important concept in viticulture and enology. It summarizes the effect of the environment on vine physiology and grape production as regulated by cultural practices (van Leeuwen and Seguin, 2006). This concept also states that origin determines the final characteristics and typicity of wines because of the peculiarity of interactions in each particular agroecosystem (OIV, 2010). The concept describes a form of agricultural management, adaptable to all plants, which must be considered an interesting alternative to intensive farming as it respects traditional practices, the products and the environment, as well as consumer pleasure. All aspects of the ecosystem play a role and are accounted for (van Leeuwen and Seguin, 2006). The application of this concept therefore requires an enhanced knowledge of site-specific ecophysiological relationships.

The understanding of grapevine water dynamics is therefore crucial to optimize vineyard management, and models can be used to summarize the complex relationships between the plant, the soil, and the climate. Furthermore, model predictions can be used to predict future trends as well as for daily practical purposes in production contexts.

Except for pest and disease control, or grapevine phenology, modeling approaches are not frequently used in viticulture, as opposed to other trees or field crops (for a review of available models for grapevine see Moriondo et al., 2015). Frequently, non-vine specific, multicrop models are used in viticulture to simulate soil water balance in vineyards, and then grapevine growth and yield. Examples are the use of SWAP (Bonfante et al., 2015), STICS (Cola et al., 2014), CropSyst (Pallas et al., 2011), and HYDRUS (Maxwell et al., 2016) models, or the use of crop coefficients, K_c_ (Fandiño et al., 2012).

Few models have been specifically developed for grapevine; examples are modeling of nitrogen dynamics in vineyards (NVINE, Nendel and Kersebaum, 2004), grape canopy structure and light interception (Louarn et al., 2008; Iandolino et al., 2013), grapevine phenology (Parker et al., 2011). However, to the best of our knowledge, only one model has been specifically developed to evaluate water balance in vineyards. This model goes back to the work of Riou et al. (1989) for the radiation partitioning and interception module, further extended by Lebon et al. (2003), to include a soil WBM, then by Celette et al. (2010), to account for the presence of cover crops. The model was last updated by Hofmann et al. (2014), who modified the radiation module and extended the application to sloped vineyards. This model, referred here as WBM, is widely used in both research (Pellegrino et al., 2006; Gaudin and Gary, 2012; among others) and production.

In general, models used in viticulture to simulate grapevine water balance are process-based; they formally describe and try to link already known physiological and environmental processes. These models need to be accurately parametrized and tend to increase in complexity in order to suit all possible real-world conditions. Specifically, the WBM needs an accurate, on-site assessment of the SWHC, also called TTSW in viticulture (as defined by Ritchie, 1981; see Brillante et al., 2015b for review and comparison with other SWHC estimation methods), which in turn requires the measurement of grapevine water status. Furthermore, the WBM does not directly predict grapevine water status but estimates it indirectly from the simulated FTSW, which has been found to be related to both stomatal conductance (Lebon et al., 2003), Ψ_pd_ (Pellegrino et al., 2005) and sap flow (Hofmann et al., 2014). A specific calibration is required for each site in order to improve WBM prediction performance.

The work presented here is different in that it empirically starts from grapevine water stress data and uses a cutting edge machine-learning approach to learn and describe their pattern as a function of environmental variables. Specifically, it seeks to directly predict leaf water potentials, commonly used to evaluate plant water status and to improve irrigation management, as a function of macroscopic soil properties and widespread meteorological observations. This work also takes advantage of the empirical approach, which does not require a previously defined form to link inputs and outcomes, to describe relationships between soil, climate and grapevine water status. Model performances are assessed by canonical practices (cross-validation, test-set) and by comparison with carbon isotope discrimination of sugars at harvest, δ^13^C (Farquhar et al., 1982, 1989), which is a continuous integrator of grapevine water status, during the veraison-harvest period (Gaudillère et al., 2002; de Sousa et al., 2005).

The aim of this research was (i) to explore the potentialities of a machine-learning algorithm to develop robust predictive models, easy to transfer in production contexts, to evaluate past and future grapevine water stress and (ii) to understand how easy-to-measure environmental factors affect grapevine water status at the local scale.

## Materials and Methods

### Experimental Field Site and Plant Material

This study was carried out over 3 years (2011–2013) in a commercial vineyard (Domaine Louis Latour, N47.071992, E4.855993, Aloxe-Corton, Burgundy, France). Eight experimental plots were selected and labeled in alphabetical order (A–H) from the top (325 m) to the bottom of the hill (267 m). Plots were 7 m × 7 m squares containing 49 grapevines (*Vitis vinifera* L. cv Chardonnay B.) grafted on SO_4_ rootstock (interspecific cross between *Vitis riparia*, Michx. and *Vitis berlandieri*, Planch.). Grapevines were between 20 and 30 years old and planted at a spacing of 1 m (between plants) × 1 m (between rows). Vines were Guyot pruned and trained in a vertical-shoot-position trellis system with the first training wire at 0.5 m and the fruiting cane trimmed at 1.20 m. Grapevine rows were oriented north–south.

At the beginning of the study, soil samples were collected at 0.1-m intervals down to 1-m depth in a trench located in the middle of each plot and analyzed to determine soil texture and gravel content. Soil properties averaged over 0–1 m depth are presented in **Table 1**; a detailed description including a larger set of soil properties can be found in Brillante et al. (2014).

**Table 1:** Summary of soil properties in the experimental field site.

Plot	Slope (%)	Gravel (%)	Texture (USDA)	Gravel (class)	Slope (class)
A	20.6	24.2	Loam	High	Steep
B	28.5	36.2	Loam	High	Steep
C	22.1	14.5	Loam	Low	Steep
D	6.2	23.9	Clay-loam	High	Mild
E	9.1	8.2	Clay-loam	Low	Mild
F	6.2	10.3	Clay-loam	Low	Mild
G	6.6	22.9	Clay-loam	High	Mild
H	4.1	26.4	Loam	High	Mild


*Slope was measured with a differential GPS and expressed in percent (change in elevation over a 100-m distance). Texture and gravel content were computed by averaging data measured at approximately 0.1-m intervals over a 1-m depth in each plot. The USDA triangle was used to classify the texture. Gravel content is expressed in percent per volume. The right side of the table shows the data in the categorical classes as used in models.*

### Meteorological Data

Meteorological data were obtained from an on-site weather station for 2012 and 2013 and from a commercial station used by grape growers and located in proximity of the study site for 2011. Both stations measured minimum and maximum temperature and rainfall. To model the relationship between climate data and plant water stress, cumulative rainfall over 7 and 14 consecutive days prior to leaf water potential measurement, and daily temperatures (minimum, maximum and computed mean) collected on the same day of measurement were used. Rainfall and temperature trends in 2012 and 2013 are shown in Brillante et al. (2016a).

### Plant Physiological Measurements

#### Leaf Water Potentials

Predawn (Ψ_pd_; Scholander et al., 1965) and Ψ_stem_ (Begg and Turner, 1970, and Choné et al., 2001 for grapevine) were monitored weekly in 2012–2013 and every 10 days in 2011, with a pressure chamber (PMS Instruments Inc., Albany, OR, USA), from bunch closure to harvest in 2012–2013 and from veraison to harvest in 2011. Eight leaves were randomly sampled in the fruit area of different grapevines (one leaf per plant) for Ψ_pd_ and twelve for Ψ_stem_; for Ψ_stem_, leaves were placed inside plastic bags covered with aluminum foil before measurement. The sampled grapevines were selected randomly and varied at each measurement. The order of testing between the eight experimental plots was randomized to avoid bias from measurement time. Both leaf water potentials were performed the same day (time lag <24 h). Values are expressed in MPa. More than 2000 Ψ_stem_ and more than 1500 Ψ_pd_ measurements were performed. Ψ_stem_ integrals were computed to allow comparison with δ^13^C, Ψ_stem_ being discrete in time while δ^13^C is continuous. This method has been proposed first by Meyers (1988), and compared first to δ^13^C by de Sousa et al. (2005). A value of 0 MPa was used as baseline in integral computation; real values were used (not absolute values).

#### Carbon Isotope Composition of Sugars, δ^13^C

Photosynthetic δ^13^C was measured on sugars in mature grapes, following the protocol described in Gaudillère et al. (2002) and van Leeuwen et al. (2010). Three 100-berry composite samples were collected from 16 randomly selected grapevines (3 samples × 8 blocks × 2 years) and isotopic analyses were performed in triplicate on a Vario Micro Cube elemental analyzer coupled in a continuous flow mode to an isotope ratio mass spectrometer (IsoPrime, Elementar). USGS40 (IAEA, Vienna) was used as an internal standard (δ^13^C_PDB_ = -26.2 ± 0.1‰). δ^13^C values are reported in parts per thousand (‰) relative to the Vienna Pee Dee Belemnite (VPDB) international reference.

### Statistical Analysis

A GBM (Friedman, 2001) with trees as base learners was used for modeling (see Brillante et al., 2015a for further details in a grapevine case study; Hastie et al., 2009 as reference text for a good introduction; and Elith et al., 2008 for a primer in ecology). The model was tuned by using 25 repetitions of ten-fold cross-validation, which were also used to assess model performance. The model was fitted on the data obtained in 2012 and 2013, while the dataset from 2011 was used as “real life” test-set, with less reliable commercial meteorological data locally used by farmers, obtained from a station in proximity (1 km), but not on-site. Multicollinearity was tested and stayed low among predictors (the higher between 7 and 14-day cumulative rainfall stays at 0.47, Kendall correlation). Meteorological data entered the model once computed in the way described in Section “Meteorological Data” above, while the soil data (slope, texture and gravel content) entered the model as categorical data, according to the groups described in **Table 1**. The statistical analysis was run in R using the GBM package (Ridgeway, 2013).

## Results

### Soil Properties

Soil sample analysis showed that plots A, B, and C had a steeper slope (higher than 20%), while plots D, E, F, G, and H had a milder slope (lower than 10%) (**Table 1**). Texture differences between plots were small, once averaged over the 1-m soil depth, and corresponded to loam (A, B, C, and H) or clay-loam (D, E, F, and G) classes. Gravel content showed a large range of variation (from 8 to 36% in volume), with plots A, B, D, G, and H >20% and plots C, E, and F <15%.

### Modeling of Plant Water Stress as a Function of Climate, Topography and Soil Properties

#### Solar Noon Stem Water Potential

Grapevine water stress ranged from low to moderate with considerable variation in measured Ψ_stem_, which ranged from -1.05 MPa to -0.24 MPa (**Table 2**). Ψ_stem_ was modeled as described in M&Ms, including as predictors maximum temperature, cumulative rainfall in the previous 7 and 14 days, slope, gravel content and soil texture (**Table 1**). Descriptive statistics for predictors and outcomes are presented in **Table 2**.

**Table 2:** Descriptive statistics for grapevine water status and meteorological data.

Variable	Minimum	Maximum	Mean	Median
Ψ_stem_ (MPa)	-1.05	-0.24	-0.58	-0.54
Ψ_pd_ (MPa)	-0.62	-0.03	-0.19	0.17
δ^13^C (‰)	-27.95	-26.33	-27.18	-27.24
Max temperature (°C)	16.50	32.60	26.14	26.60
Cumulative rainfall in the 7 previous days (mm)	0.00	47.40	14.43	8.40
Cumulative rainfall in the 14 previous days (mm)	4.60	96.40	29.82	17.80


**FIGURE 1:**
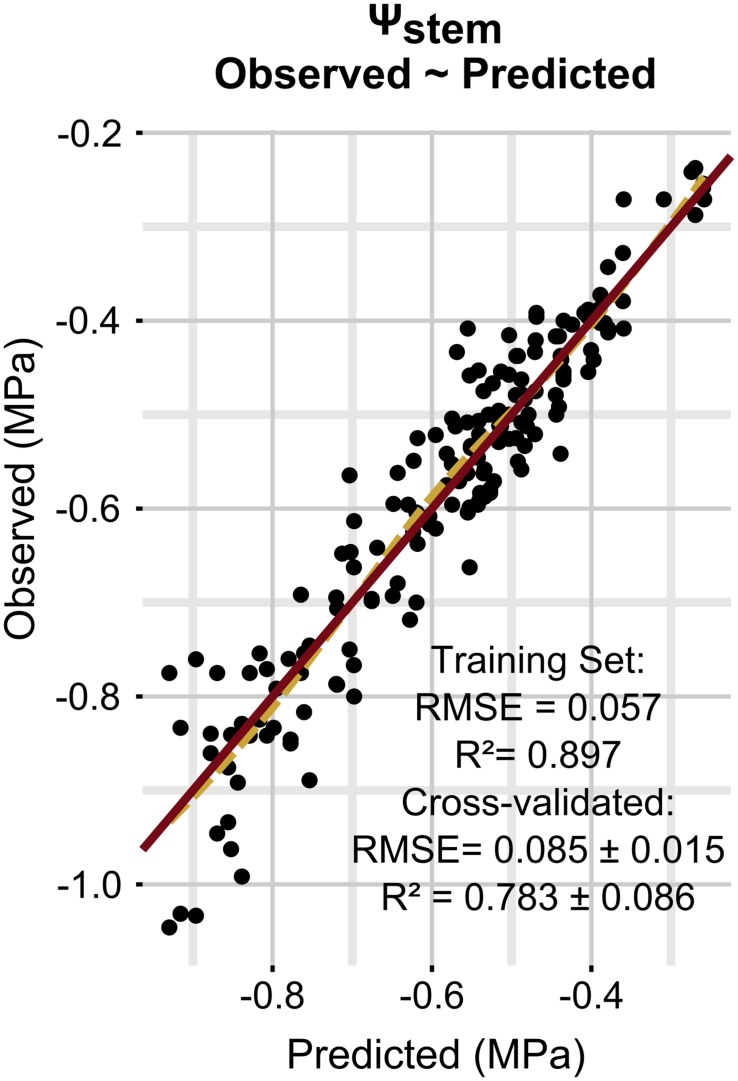
**Relationships between observed and predicted Ψ_stem_ data (training dataset is shown).** The solid line is a line with slope 1 and intercept 0, while the dashed line is a loess fitted to the data.

Model performances are presented in **Figure 1**. Ψ_stem_ was predicted with a RMSE of 0.085 ± 0.015 MPa and a coefficient of determination (*R*^2^) of 0.783 ± 0.086 on simulated new data as evaluated by cross-validation. The regularization process gave the best results with 1000 trees having eight splits, a shrinkage equal to 0.03, bag fraction set to 0.5, and minimum 10 data points in final tree nodes.

All eight predictors included in the model had non-null influence (i.e., each predictor contributed to the prediction of the outcome), and maximum temperature was the one with the highest relative contribution (**Table 3**).

**Table 3:** Relative influence of predictors in the solar noon stem leaf water potential (Ψ_stem_) model (scaled so that the sum of all relative contributions is 100).

Predictors in Ψ_stem_ model	Relative influence (%)
Maximum temperature	28.21 ± 0.82
Cumulative rainfall in the 7 previous days	25.77 ± 1.06
Cumulative rainfall in the 14 previous days	24.16 ± 0.97
Slope	12.92 ± 0.55
Gravel content	6.42 ± 0.46
Soil texture	2.54 ± 0.17


**FIGURE 2:**
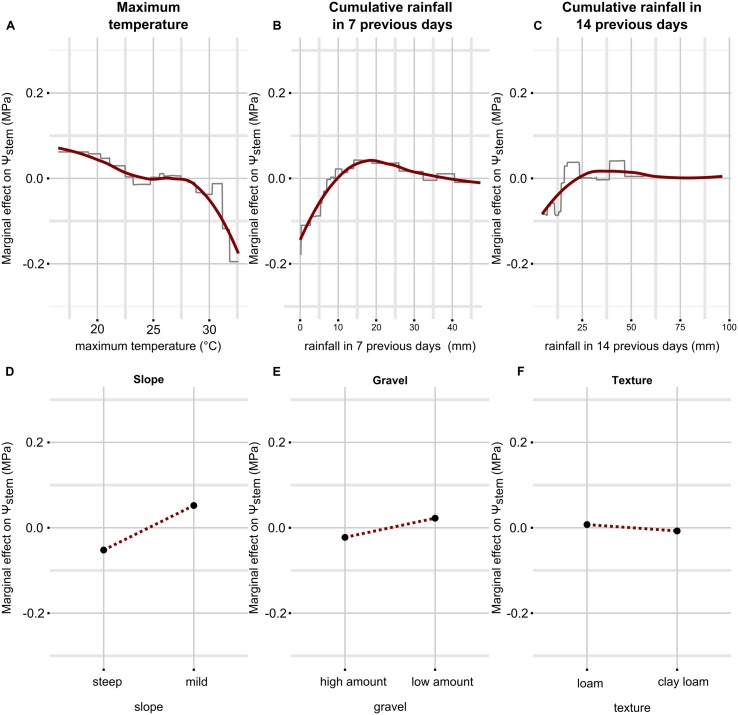
**Partial dependence plots for each predictor in the Ψ_stem_ model.** Only simple relationships (no interactions) are shown. They have been obtained by predicting Ψ_stem_ while fixing all predictors to their mean value except the one in question, which was allowed to finely vary across the range of observed data. On the *y* axis there is the marginal effect on Ψ_stem_, i.e., when it has a 0 value, Ψ_stem_ is estimated to its mean by the predictor in question, when it has a value different from 0, Ψ_stem_ is estimated higher or lower than its mean by the corresponding value. On the *x* axis there is the range of observed data for the predictor in question. The essential relationships between predictor and outcome are captured in a smoothed fashion by the red line which is a loess applied to the partial prediction data. The gray lines are original functions as based on the trees used in the models. When the predictor is discrete (as for soil properties), the function shown in the partial dependence plots is also discrete and has a single value for each level of the predictor (soil properties are binary here, then two values). See text for detailed description. Plot **(A–F)** shows partial dependence plots between predictor and outcome: **(A)** for temperature, **(B)** for cumulative rainfall in 7 previous days, **(C)** for cumulative rainfall in 14 previous days, **(D)** for slope, **(E)** for gravel, **(F)** for texture.

To understand the nature of the dependence between outcome and predictors, it is valuable to look at the partial dependence plots (**Figure 2**). These plots give a graphical summary of the relationship between predictors and the outcome (Ψ_stem_), in average conditions of all variables (set constant to their mean) except the predictor variable in question. Attention has to be paid to rainfall, because two model parameters are derived from this variable. Therefore, the effect of rainfall in 7 days takes into account 30 mm of rain in 14 days (which is the recorded mean); conversely, the effect of rainfall in 14 days takes into account 14 mm in the week before measurement. When maximum temperature ranges between approximately 22 and 28°C, the effect on Ψ_stem_ is null; when maximum temperature decreases below 22°C, Ψ_stem_ increases; conversely, when maximum temperature increases over 28°C, Ψ_stem_ decreases steeply (**Figure 2A**). Cumulative rainfall in the previous 7 (**Figure 2B**) and 14 (**Figure 2C**) days both affect Ψ_stem_ predictions in a similar manner, but obviously with different absolute values: Ψ_stem_ decreases when rainfall is below 10 mm (25 mm) in the 7 (14) days before leaf water potential measurement. From 10 to 15 mm, cumulative rainfall during the 7 previous days induces a rise in Ψ_stem_, then this positive effect gradually decreases until it no longer positively affects Ψ_stem_ prediction with respect to the mean. Over 25 mm, cumulative rainfall 14 days before measurements does not affect substantially Ψ_stem_ estimates with respect to the mean within the model. The soil characteristics included in the model suggest that when the slope is steeper, the gravel content is higher and the soil texture is richer in clay, Ψ_stem_ decreases, while it increases when the slope is mild, the gravel content is lower and the soil is loamy.

**FIGURE 3:**
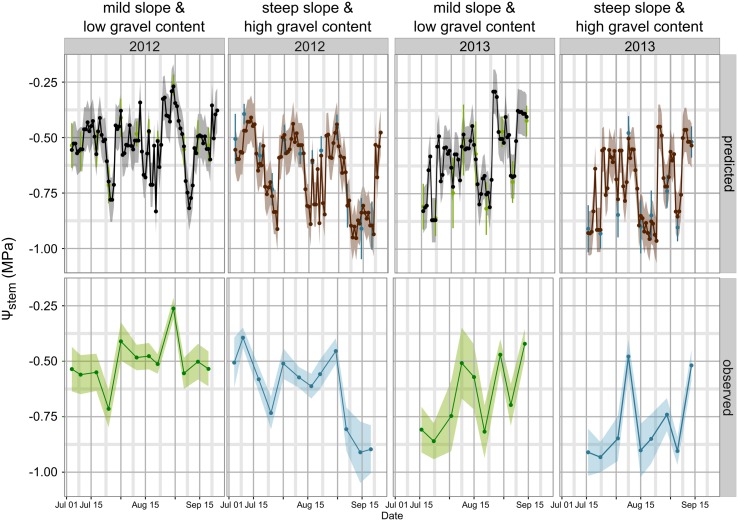
**Model simulations for Ψ_stem_ in 2012 and 2013 between bunch closure and harvest.** Veraison was approximately 15–16 August in both years. Two extreme scenarios are presented: (1) mild slope and low gravel content (clay-loam texture): lower water stress; and (2) steep slope and high gravel content (loam texture): higher water stress. For observed data the standard deviation of measurements is the shaded area in lower panel trends, and error bars for green and blue points in the upper panel. For predicted trends, the shaded area is equal to 0.1 MPa, the maximum estimated RMSE error in cross-validation (upper panel).

**Figure 3** simulates the Ψ_stem_ trend in 2012 and 2013 (the training vintages) for the two most extreme cases in the dataset: steep slope and high gravel content (loam texture), and mild slope and low gravel content (clay-loam texture). As shown in **Figure 2F**, the loam texture indicates a higher Ψ_stem_ than the clay-loam texture, but a combination of all factors inducing water stress was not present in the experimental site (**Table 1**) and most important were retained. The model always predicts Ψ_stem_ within the standard deviation, which is also summarized in **Table 4**, and can be compared to the cross-validated RMSE error of the model: 0.085 ± 0.015 (**Figure 1**).

**Table 4:** Descriptive statistics of standard deviation for measured (observed) Ψ_stem_.

	Whole dataset (n = 168)	Steep and high gravel	Mild and low gravel
Mean (MPa)	0.078 ± 0.033	0.082 ± 0.027	0.090 ± 0.03
Min (MPa)	0.019	0.019	0.033
Max (MPa)	0.23	0.15	0.23


*Mean, minimum and maximum are shown and computed on data grouped by date of measurement (12 leaves per site per date; 21 dates; whole dataset: 8 sites; steep and high gravel content: 2 sites; mild and low gravel content: 2 sites). Cross-validated error of the model is 0.085 ± 0.015.*

**FIGURE 4:**
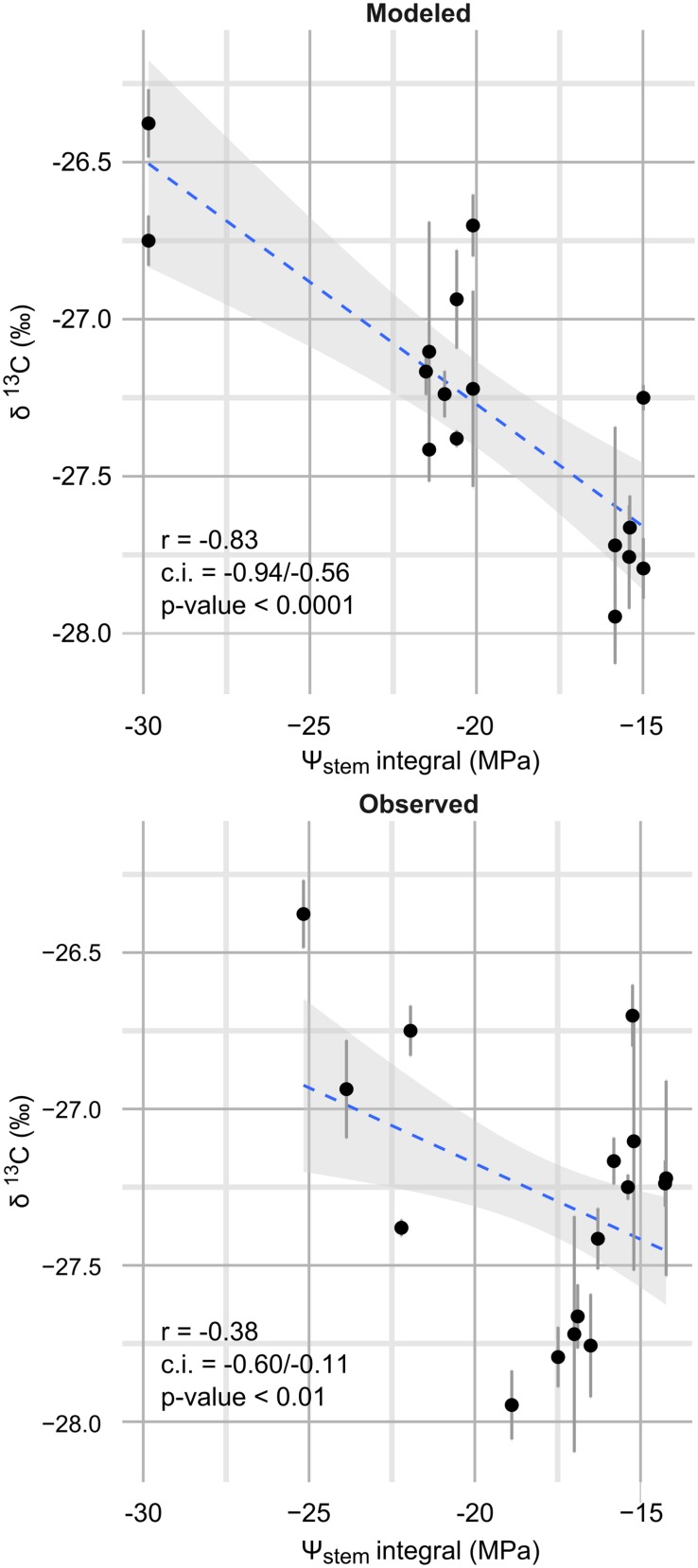
**Correlation between Ψ_stem_ integrals and δ^13^C (‰).** Ψ_stem_ integrals were measured on all eight sites in 2012 and 2013 between veraison (2012-08-17; 2013-08-15) and harvest (2012-09-20; 2013-09-13). Upper panel: Ψ_stem_ integrals measured on the daily Ψ_stem_ trend as estimated by the model; lower panel: Ψ_stem_ integrals measured on the measured trend (observed), at weekly time scale. Error bars are standard deviation of δ^13^C biological replicates. The blue line is the best fit (OLS regression); the shaded area shows the confidence intervals for this line.

**FIGURE 5:**
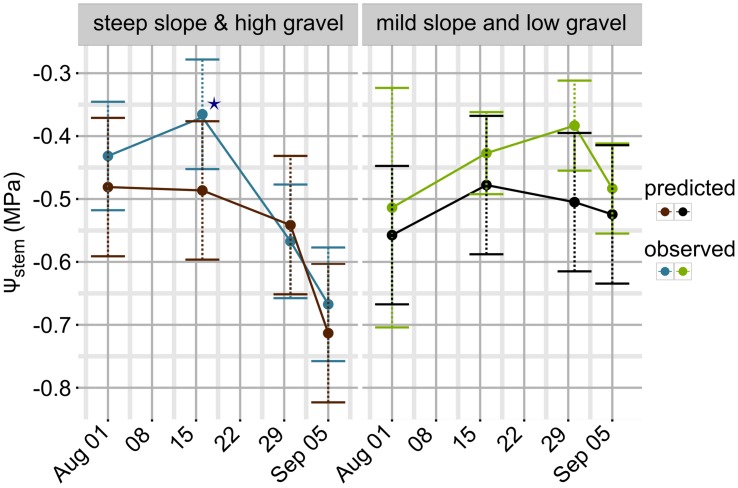
**Observed and predicted Ψ_stem_ trends in 2011 (test-set and different meteorological data).** Error bars are mapped to standard deviation for observed data points (blue: steep slope and high gravel content; green: mild slope and low gravel content). For predicted values, error bars are equal to 0.11 MPa, i.e., the RMSE evaluated on the test-set. ★ indicates missing data for plot A and B on 2011-08-16 (both steep slope and high gravel content, **Table 1**); missing data were replaced by the mean of the whole dataset for this date.

Observed data points were measured weekly, while model predictions are shown at a daily time step (**Figure 3**). Departures from the observed trends are present in the simulation, and generally the model predicts low potential values more frequently. As an example, see around 2012-15-08 for the steep slope scenario, or compare the beginning of September 2012 for the mild slope scenario. Are those daily simulations correct? An answer could be obtained from the comparison of the Ψ_stem_ integral computed on model simulations from all eight sites with the δ^13^C measured on must at harvest. The δ^13^C integrates grapevine water stress over the veraison-harvest period and is therefore a continuous estimator of grapevine water stress (Gaudillère et al., 2002; de Sousa et al., 2005), as is also the Ψ_stem_ integral (Meyers, 1988; de Sousa et al., 2005). **Figure 4** shows that the refinement of the trend at a daily time step (modeled) greatly improves the correlation (*r* = -0.83, *p* < 0.0001) with respect to the observed weekly time step (*r* = -0.38, *p* < 0.01). Daily simulations are therefore coherent. Furthermore, the model predicts Ψ_stem_ within standard deviation of measurements even in the 2011 vintage (test-set, not used to build the model and using a different meteorological source) (**Figure 5**). The model predicted Ψ_stem_ in 2011 with an RMSE error of 0.11 MPa.

#### Predawn Leaf Water Potential

Absolute Ψ_pd_ varied less than Ψ_stem_, ranging from -0.62 MPa to -0.03 MPa, indicating a null to moderate-severe water deficit. Therefore, Ψ_pd_ indicated a lower water deficit for grapevine than Ψ_stem_. The correlation with Ψ_stem_ was significant (*p* < 0.001, df = 142) but was also greatly scattered (*r* = 0.29). Using an approach similar to the one previously used for Ψ_stem_, a model for Ψ_pd_ was also developed. All the predictors used to develop the Ψ_stem_ model were also used for the Ψ_pd_ model (**Tables 1** and **2**), but minimum temperature was used instead of maximum temperature, because Ψ_pd_ is measured at the end of the night when minimum temperatures are generally recorded. However, mean and maximum temperatures were also tested as predictors in the model but minimum temperature gave better results in the cross-validation procedure. Minimum temperature varied less than maximum temperature (∼60% less) and ranged from 9.3°C to 19.3°C.

**FIGURE 6:**
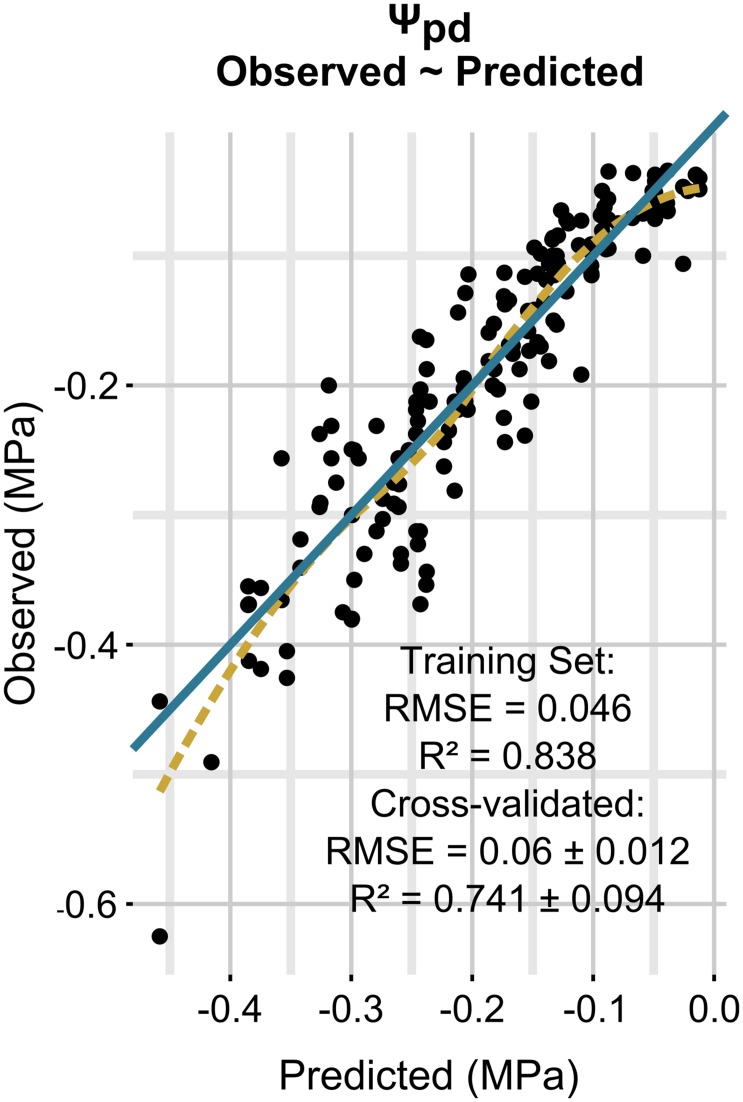
**Relationships between observed and predicted Ψ_pd_ data (training dataset is shown).** The solid line is a line with slope 1 and intercept 0, while the dashed line is a local polynomial regression (loess) fitted to the data.

Model performances are shown in **Figure 6**. The model predicts Ψ_pd_ with a RMSE of 0.06 ± 0.012 MPa and a *R*^2^ of 0.741 ± 0.094, as evaluated by cross-validation. Measurement standard deviation (observed) was 0.049 ± 0.026 MPa. The regularization process gave the best results with 1650 trees having two splits, a shrinkage equal to 0.035, a bag fraction set to 0.5, and 10 data points in final tree nodes. All eight predictors included in the model had non-null influence, and minimum temperature was the one with the highest relative contribution, although very similar to cumulative rainfall in the 14 previous days (**Table 5**). Cumulative rainfall in the 7 previous days had a lower importance in the model than rainfall in the 14 previous days; this was also a significant difference with respect to the Ψ_stem_ model, where both rainfall predictors had similar importance. As for Ψ_stem_, the soil properties had a lower influence in the model than the climate, and their rank was the same as for Ψ_stem_. The relative values of predictor contribution were also very similar in both models.

**Table 5:** Relative influence of predictors in predawn leaf water potential (Ψ_pd_) model (scaled so that the sum of all relative contributions is 100).

Predictors in Ψ_pd_ model	Relative influence (%)
Minimum temperature	31.02 ± 0.97
Cumulative rainfall in the 14 previous days	29.99 ± 0.90
Cumulative rainfall in the 7 previous days	19.1 ± 0.81
Slope	12.13 ± 0.42
Gravel content	5.28 ± 0.31
Soil texture	2.48 ± 0.22


**FIGURE 7:**
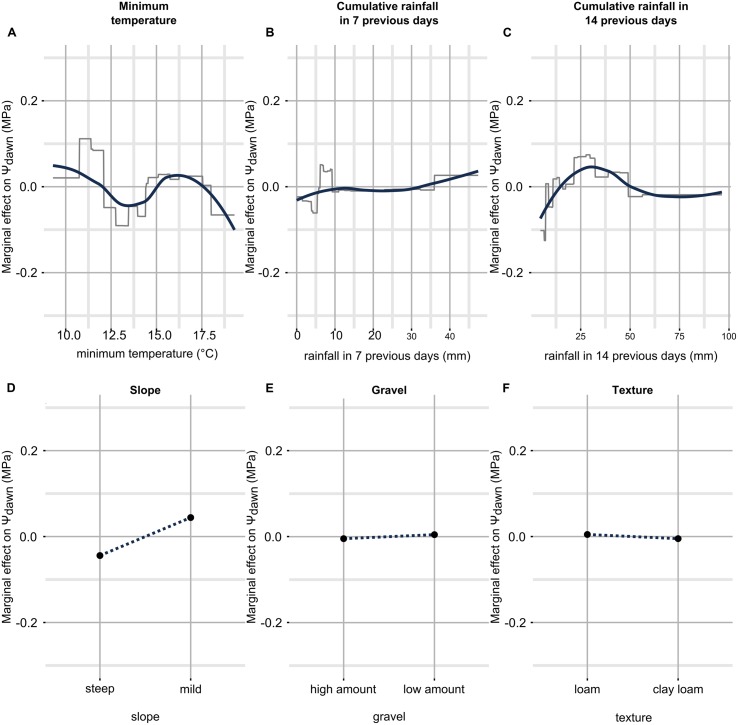
**Partial dependence plots for each predictor in the Ψ_pd_ model.** Only simple relationships (no interactions) are shown. They have been obtained by predicting Ψ_pd_ while fixing all predictors to their mean value except the one in question, which was allowed to finely vary across the range of observed data. On the *y* axis there is the marginal effect on Ψ_pd_, i.e., when it has a 0 value, Ψ_pd_ is estimated to its mean by the predictor in question, when it has a value different from 0, Ψ_pd_ is estimated higher or lower than its mean by the corresponding value. On the *x* axis there is the range of observed data for the predictor in question. The essential relationships between predictor and outcome are captured in a smoothed fashion by the red line which is a loess applied to the partial prediction data. The gray lines are original functions as based on the trees used in the models. When the predictor is discrete (as for soil properties), the function shown in the partial dependence plots is also discrete and has a single value for each level of the predictor (soil properties are binary here, then two values). See text for detailed description. Plot **(A–F)** shows partial dependence plots between predictor and outcome: **(A)** for temperature, **(B)** for cumulative rainfall in 7 previous days, **(C)** for cumulative rainfall in 14 previous days, **(D)** for slope, **(E)** for gravel, **(F)** for texture.

Partial dependence plots for the Ψ_pd_ model are shown in **Figure 7**. The relation between temperature and Ψ_pd_ did not have a clear structure as the one observed for Ψ_stem_ (**Figure 7A**). A general decrease in Ψ_pd_ with increasing temperature is observed, but is gentle and also noisy. The noise can be related to the error in the model predictions, in anomalous data points with high leverage, but also to variations of other parameters (e.g., the vapor pressure deficit, VPD) to which air temperature is more or less related and which are not taken into account by the model. Considering rainfall (**Figures 7B,C**), if the amount of rain in the last 7 (14) days is lower than 5 mm (10 mm), Ψ_pd_ decreases, while if the amount of rain in the last 7 (14) days is between 5 and 10 mm (25–50 mm), Ψ_pd_ increases. When precipitations in the 7 previous days are more abundant than 10 mm, the effect within Ψ_pd_ prediction is null. Ψ_pd_ increases only when heavy rains were observed (more than 30 mm in a week). Surprisingly and conversely, when cumulative rainfall 2 weeks before leaf water potential measurement increases over 50 mm, a slight decrease in Ψ_pd_ is observable. The effect of soil properties on Ψ_pd_ (**Figures 7D–F**) was similar to that for Ψ_stem_; they acted in the same directions in both models.

## Discussion

The study of the relationship between plants and their environment, especially in relation to biotic and abiotic stresses, has acquired a renewed importance in recent years because of the increased awareness about climate change. Accurately modeling and predicting physiological responses of plants to these stresses, such as water stress, has a strategic importance to increase producer awareness in a rapid and cost effective way, and to allow adaptation of agronomic practices to future conditions.

As opposed to previous works on the WBM of vineyards (Lebon et al., 2003; Celette et al., 2010; Hofmann et al., 2014), this work did not have the aim to build a framework extensible to all vineyards. This work instead proposed a strategy to modeling leaf water potentials from macroscopic soil and climate data, which are easily available to both scientists and grape growers. It is a straight-forward approach to predict plant water status at a daily time step and can therefore be used to simulate future and past local scenarios as well increase the time resolution of this traditional measurement. By modeling highly non-linear relationships as those shown in **Figures 2** and **4**, as well non-linear interactions between predictors (not shown), the used machine-learning approach allowed to obtain very low errors in the direct estimation of leaf water potentials, probably the most widespread measurements of plant water stress in commercial vineyards. The reported errors are comparable to measurement standard deviation (**Table 4**). This is the first time that grapevine leaf water potentials are directly modeled by a machine-learning approach, and the first time for Ψ_stem_ itself. Here a GBM was used, but other non-linear methods such as neural networks, random forests, etc. could probably be effective as well.

The proposed strategy suffers from the typical problems related to empirical approaches, as summarized by Adams et al. (2013). The main problem is that it depends on the learned data, and estimation of leaf water potentials cannot be made outside the range of values observed for each predictor (i.e., extrapolation). For the same reason the spatial scale of prediction is reduced to the scale of calibration. This problem limits the suitability to large scale simulation of climate change for which process-based models are probably more effective (Cuddington et al., 2013; Moriondo et al., 2015). A solution to this limit could be the acquisition of datasets including a great variation in predictor values, and extending the calibration area. Large datasets are also mandatory to accurately fit and interpret machine-learning methods.

Models were assessed using cross-validation, a test-set and correlation with δ^13^C. The used test-set is not very large, and can therefore be considered more as an application of the model on unseen data, than as a method to accurately assess model performances, to which cross-validation is very effective and best suited in this, and other similar cases (Hastie et al., 2009). For this reason commercial meteorological data from a different station were used. The correlation with δ^13^C, was used to evaluate the ability of the model to daily predictions of plant water status, being data used to build the model collected at a weekly time-scale. Carbon isotopic discrimination has already been proposed as a method to test models of optimal stomatal conductance in response to environmental gradients (Farquhar et al., 2002; Wright et al., 2003; Medlyn et al., 2011). Measured on grape sugar at harvest it has been proposed as an integral estimator of grapevine water status between veraison and harvest (Gaudillère et al., 2002; de Sousa et al., 2005). However, it is also related to genotype differences, nitrogen, and other environmental factors (see Cernusak et al., 2013, for a review). Because of the use made here of this measurement, those nuisance factors did not affect results: two correlations, obtained with the same δ^13^C data are compared, the scale of the study site is small, plant genotype is very similar.

Compared to the WBM, the use of raw soil and climate data to directly predict plant water stress has two advantages: (i) it allows to avoid SWHC measurements, or TTSW as commonly referred to in viticulture (here the two terms are interchangeable), which is the basis of the WBM; and (ii) it allows to directly express plant water status as a function of both soil and climate data.

More specifically:

(i) An accurate measurement of the TTSW is problematic, expensive and time consuming. TTSW needs an accurate field assessment of volumetric soil water. It cannot be inferred by a laboratory analysis of soil properties, being dependent from root uptake, and at the same time it also demands estimation of the plant water stress (Ritchie, 1981; Pellegrino et al., 2005; Brillante et al., 2015b). The accuracy of measuring devices for the estimation of soil volumetric content also causes problems. As an example, time-domain reflectometry (TDR), which is among the most used devices for this scope, commonly has an error in estimation of ±2% vol. (manufacturer values, in perfect operating conditions). The error can accumulates when estimating low and high limits of TTSW (up to ± 4% vol.), and in the worst cases finally brings to a ± 15–40% biased estimation of TTSW, which approximately ranges for grapevine between 10 and 30% vol. (Brillante et al., 2016b).
(ii) The WBM does not directly predict plant water stress but correlates it in a second step to the simulated FTSW through a bilinear function (Lebon et al., 2003), which was empirically found (Trambouze and Voltz, 2001) where the correlation between the two variables no longer exists for FTSW values higher than 0.4 (FTSW is scaled between 0 and 1). This reduces the accuracy when FTSW is above the threshold, but has allowed stomatal conductance (Lebon et al., 2003) or sap flow (Hofmann et al., 2014) to be simulated. The 0.4 value is used as a threshold of water stress, i.e., at higher FTSW values plant transpiration is not limited by soil water. However, this threshold does not correspond to a fixed transpiration value, which instead varies between sites as shown in Hofmann et al. (2014), and therefore a site specific calibration is requested. Furthermore, even if the effect of climate factors (evapotranspiration) is included in the model to simulate soil-water consumption, a direct effect on plant water status is not included. Plant water status can be affected by the climate independently from soil water, as shown in **Figure 2A** for temperature on Ψ_stem_, which can down-regulate (Greer and Weedon, 2012) or up-regulate (Greer and Weedon, 2013) gas exchanges, or as shown by Poni et al. (2009) for VPD. This can also limit the WBM in simulating climate change scenarios, especially in regions where temperature is expected to rise but rainfall evolution remains strongly uncertain (IPCC, 2014). Results in **Figure 2A** show that Ψ_stem_ appears strongly dependent on maximum temperature, which can also act here as a proxy for VPD. This merits to be verified in future studies.

It is interesting to observe differences in the effect of the rainfall in the 7 previous days on Ψ_stem_ and Ψ_pd_, as shown in the respective partial dependence plots (**Figures 2B,C**), or the respective tables for the relative influence of predictors (**Tables 3** and **5**). The Ψ_stem_ response to rainfall is clearly stronger than that of Ψ_pd_, the latter appearing as a more conservative evaluation of plant water stress. As proposed in Brillante et al. (2016a), the faster response of Ψ_stem_ to re-watering could be due to the plants’ large sensitivity during the day to water refill in the shallow layers. Indeed, lower leaf/stem water potential allows water extraction in layers where water is stored at higher tensions, among which the shallower layers, which rapidly dried because of both plant transpiration and soil evaporation. During the night, the lower tensions in plants reduce the possibilities of using the dry shallower layers and plants principally equilibrate with deeper horizons.

Slope was the main soil factor influencing grapevine water stress (**Tables 3** and **5**; **Figures 2D** and **7D**), probably because of surface runoff occurring in this condition, which depends on the rainfall intensity at a daily rate, and the soil tillage. In vineyards, a runoff threshold has been estimated at 6 mm of daily rain for bare soil and 25 mm for a cover crop (Celette et al., 2008). Soil sites in this study were both tilled and crop covered. The effect of steep slope shown in this study confirms the results by Hofmann et al. (2014) where steep sloped vineyards were reported to decrease their relative transpiration rate with increasing evaporation demand, while the effect was reduced for mild sloped vineyards.

The role of soil texture and gravel content on plant water stress has already been investigated in many studies (Gaudillère et al., 2002; van Leeuwen et al., 2004, 2009; des Gachons et al., 2005; Tramontini et al., 2013a; Bonfante et al., 2015). A recent ecophysiological review can be found in Lovisolo et al. (2016). The use of soil properties by the models presented in this study is coherent with previously cited research. The water stress experienced by grapevines in gravelly soils is predicted to be more severe than in soils with low gravel content (**Figures 2E** and **7E**), because gravel directly reduces TTSW. Models predict a higher water stress for soil with a finer texture with respect to soils with a coarser texture. This effect is related to the reduction in matrix potential of soil with increasing clay. It has also been shown that leaf ABA concentration is higher in clay rich soils, therefore determining stomatal closure and lowering transpiration (Tramontini et al., 2014). It has been proposed that anisohydric (or drought-resistant) cultivars, such as the Chardonnay (Vandeleur et al., 2009), could be more sensitive to soil characteristics, than to climate (Lovisolo et al., 2016). This could probably be true when compared to isohydric cultivars, but in this study the climate had a greater effect respect to the soil. Rootstock also plays a significant role in the adaptation of the scion to the environment (Tramontini et al., 2013b), and the SO_4_, being low-medium tolerant to drought (Lovisolo et al., 2016) presents an opposite behavior respect to Chardonnay. However, the effect of texture was reduced in this study, compared to these cited works, because differences between experimental sites were not very strong: texture averaged over the first meter ranged from loamy to clay-loamy, sandy soils were absent.

## Conclusion

Empirical models able to predict Ψ_stem_ and Ψ_pd_ with high accuracy were developed for grapevine using a machine-learning approach. Temperature appeared as a very important predictor in determining the water stress experienced by grapevine, especially at midday. In the presented models it directly affected Ψ_stem_, independently from rainfall, and then soil water. In predictive models, it can also act as a proxy for evaporation demand. The response to re-watering appeared different when considering the water stress measured at night and during the day, and close in time rainfall had more effect in the alleviation of water stress experienced at midday than at night.

To build empirical predictive models as in this study to evaluate the water stress experienced by grapevine allows reaching very good performance. It can be considered a useful strategy to simulate past and future plant water stress in field condition, at a local scale. It can also be useful to investigate ecological relationships in the vineyard and adapt cultural practices to future conditions.

## Author Contributions

LB acquired, analyzed and interpreted data, and wrote the first draft of the paper. OM, JL, and BB provided guidance on all aspects of the study, critically reviewed the paper, and contributed valuable discussion. All authors have read and approved this version of the manuscript.

## Conflict of Interest Statement

The authors declare that the research was conducted in the absence of any commercial or financial relationships that could be construed as a potential conflict of interest.

## Abbreviations

Ψ_stem_: solar noon stem water potentials
Ψ_pd_: predawn leaf water potentials
δ^13^C: carbon isotopic discrimination (here of grape sugars at harvest)
FTSW: fraction of transpirable soil water
GBM: gradient boosting machine
LOESS: locally estimated scatterplot smoothing
RMSE: root mean squared error
SWHC: soil water holding capacity (here the same of TTSW)
TTSW: total transpirable soil water
WBM: water balance model
